# Optimization of ultrasound-assisted extraction and biological activities of *Crataegus monogyna* Jacq. flowering branches using experimental design and artificial neural networks

**DOI:** 10.1039/d6ra03013k

**Published:** 2026-05-26

**Authors:** Sengul Uysal, Aleksandra Cvetanović Kljakić, Biljana Lončar, Gokhan Zengin, Ugur Cakilcioglu

**Affiliations:** a Erciyes University, Halil Bayraktar Health Services Vocational College Kayseri Turkey senguluysal@erciyes.edu.tr; b Erciyes University, Drug Application and Research Center Kayseri 38280 Turkey; c Faculty of Technology Novi Sad, University of Novi Sad Novi Sad 21000 Serbia; d Selcuk University, Department of Biology, Science Faculty Konya Turkey; e Munzur University, Pertek Sakine Genç Vocational School Pertek, Tunceli Turkey

## Abstract

*Crataegus monogyna* is widely used in traditional medicine due to its rich content of bioactive compounds. The present study aimed to evaluate the influence of extraction parameters, including solvent concentration, extraction time, temperature, and solid-to-liquid ratio, on total phenolics, total flavonoids, antioxidant DPPH (2,2-diphenyl-1-picrylhydrazyl), ABTS (2,2′-azino-bis(3-ethylbenzothiazoline-6-sulphonic acid), FRAP (ferric reducing antioxidant power), and CUPRAC (cupric reducing antioxidant capacity)), and enzyme inhibitory properties (α-amylase and tyrosinase). Furthermore, an Artificial Neural Network (ANN) model was applied to optimize the extraction conditions. The results demonstrated that extraction conditions significantly affect both chemical composition and biological activity. The optimal conditions (70% ethanol, 30 min, 45 °C, (1 : 30)) yielded extracts with high total phenolic (109.15 mg GAE per g), and total flavonoid content (70.74 mg RE per g), strong antioxidant activity (DPPH: 473.57 mg TE per g, ABTS: 537.58 mg TE per g, CUPRAC: 407.11 mg TE per g, FRAP: 393.91 mg TE per g) and notable enzyme inhibitory potential (0.53 mmol ACAE per g against α-amylase and 75.00 mg KAE per g against tyrosinase). The ANN model showed excellent predictive performance, confirming its suitability for modeling and optimization of complex extraction systems. The present study provides comprehensive insights into optimized ultrasound-assisted extraction methods for the recovery of maximum bioactive compounds and biological activities (antioxidant and enzyme inhibition effect) from *C. monogyna* flowering branches using experimental design and ANN model. These findings provide a reliable basis for the development of functional and pharmaceutical products.

## Abstract 2

The optimal extraction conditions (70% ethanol, 30 min, 45 °C, 1 : 30 ratio) resulted in extracts with a well-balanced profile of bioactive compounds and biological activities.

## Graphical abstract

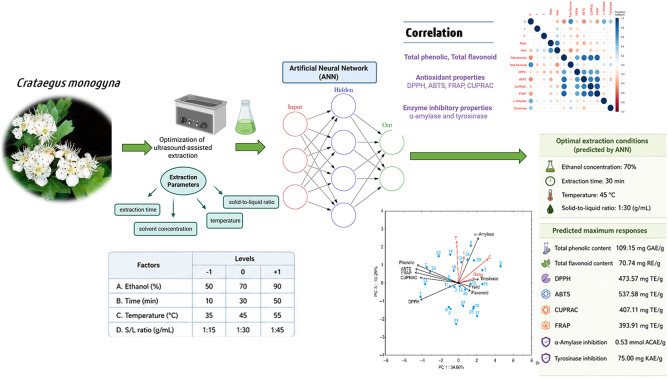

## 1. Introduction

Plant-derived natural products remain an important source of bioactive compounds with significant pharmaceutical and biomedical potential. For centuries, medicinal plants have been used in traditional medicine systems worldwide as therapeutic agents for the treatment of various acute and chronic diseases.^1^ Modern phytochemical and pharmacological studies have identified numerous biologically active constituents from plants and clarified many of their mechanisms of action, supporting their increasing integration into contemporary pharmaceutical and medical applications.^2^

Among medicinal plants, *Crataegus monogyna* Jacq. (hawthorn) has attracted considerable attention due to its wide range of biological activities and extensive use in both traditional and modern phytotherapy. As a member of the Rosaceae family,^3^*C. monogyna* is widely distributed across various regions of Asia, Africa, and Europe.^4^ It is a well-known source of biologically active compounds, including polyphenols such as chlorogenic acid, epicatechin, isoquercetin, and procyanidins, as well as triterpene acids such as oleanolic and ursolic acids.^5–7^ Owing to this complex phytochemical composition, *C. monogyna* exhibits numerous pharmacological properties, including cardioprotective, hepatoprotective, antidiabetic, antioxidant, and anti-inflammatory effects.^8–13^ In traditional medicine, different parts of the plant have been used for the treatment of cardiovascular disorders, insomnia, respiratory diseases, gastrointestinal ailments, and urinary disorders.^14–16^

The biological activity and chemical composition of plant extracts are strongly influenced by the extraction procedure and operating conditions. Extraction efficiency depends on several parameters, including solvent composition, temperature, plant-to-solvent ratio, and extraction time.^17^ In recent years, modern extraction techniques have been increasingly applied to improve extraction yield and reduce processing time and energy consumption. Among them, ultrasound-assisted extraction (UAE) has emerged as an efficient and environmentally friendly technique due to its simplicity, rapid extraction, and suitability for both laboratory and industrial applications.^18^ However, despite its advantages, UAE requires careful optimization of process conditions. Ultrasonic treatment may induce cavitation effects, local temperature increases, free radical formation, and degradation of thermolabile compounds, which can negatively affect extraction efficiency and the stability of target bioactive constituents.^19,20^ Therefore, optimization of extraction parameters is essential for achieving high recovery of biologically active compounds while preserving their functional properties.

Several studies have investigated the optimization of extraction procedures for *C. monogyna*. Martín-García *et al.* (2021) optimized ultrasound-assisted extraction conditions for *C. monogyna* leaves using a Box–Behnken design in order to maximize phenolic content.^21^ Mehmood *et al.* (2024) examined the reaction parameters for the biosynthesis of silver nanoparticles using *C. monogyna* leaf extract.^22^ In addition, Shortle *et al.* (2013) reported optimal supercritical fluid extraction conditions for leaf, flower, and berry extracts of *C. monogyna*.^23^ Nevertheless, studies focused on flowering branches of *C. monogyna*, particularly regarding enzyme inhibitory activities and advanced predictive modeling approaches, remain very limited.

Therefore, the aim of this study was to investigate the effects of extraction parameters on extraction yield, total phenolic content, and biological activities of *C. monogyna* flowering branch extracts obtained by ultrasound-assisted extraction. In addition, artificial neural network (ANN) modeling was applied to evaluate and predict the influence of extraction conditions on antioxidant activity and inhibition of enzymes associated with diabetes mellitus (α-amylase) and skin disorders (tyrosinase). To the best of our knowledge, this is the first study examining the influence of extraction conditions on α-amylase and tyrosinase inhibitory activities of *C. monogyna* flowering branch extracts using ANN-based modeling.

## 2. Materials and methods

### 2.1. Plant materials

*Crataegus monogyna* Jacq. was collected in Tunceli (Pertek Derebaşı mah, Experimental Animal Research Center around) on May. 2024. The plant material was identified by Dr Uğur Çakılcıoğlu, and the voucher specimen was deposited in the Herbarium of Munzur University (voucher number: UC-24-47).

The collected material (flowering and leafy branches) was shade-dried at room temperature for ten days and subsequently ground using a laboratory mill. Ultrasound-assisted extraction (UAE) was performed using ethanol at different concentrations (50%, 70%, and 90%), over three extraction times (15 min, 30 min, and 45 min). In addition, three extraction temperatures (30 °C, 45 °C, and 60 °C) and plant: solvent ratios (15, 30, and 45) were applied. The obtained extracts were filtered, and the solvent was evaporated under vacuum at 40 °C. All extracts were stored at +4 °C until further analysis.

### 2.2. Total bioactive compounds (total phenolic and flavonoid content) and biological activities (antioxidant and enzyme inhibition)

The total phenolic and flavonoid contents were determined using spectrophotometric methods: the Folin–Ciocalteu and AlCl_3_ assays, respectively. The results were expressed as gallic acid equivalents (mg GAE per g extract) for total phenols and rutin equivalents (mg RE per g extract) for total flavonoids.^24^

Antioxidant capacity was evaluated using radical scavenging assays (DPPH and ABTS) and reducing power assays (CUPRAC and FRAP).^24^ The antioxidant activities were expressed as Trolox equivalents (mg TE per g extract). The enzyme inhibitory activities against tyrosinase and α-amylase were also evaluated. Tyrosinase inhibition was expressed as kojic acid equivalents (mg KAE per g extract), while α-amylase inhibition was expressed as acarbose equivalents (mmol ACE per g extract).^24^ Detailed procedures are given in the SI.

### 2.3. Statistical analysis

The experimental data were statistically analyzed using StatSoft Statistica 10.0® software. To visualize the complex interactions between variables, a color correlation plot was generated in R software v.4.0.3 (64 bit), with circle diameter and color intensity used to represent the correlation coefficients. A blue-to-red color gradient was applied, where blue shades denote a positive correlation and red shades signify a negative correlation among the evaluated parameters.^25^ To evaluate the significance of the experimental data, a one-way analysis of variance (ANOVA) was conducted and for the identification of statistically significant differences between observed parameters, Tukey's *post-hoc* test was employed at a confidence level of 95% *p* < 0.05. All measurements were performed in triplicate, and the results are presented as means ± standard deviation.

### 2.4. ANN modelling

Artificial Neural Network (ANN) models were developed using a multi-layer perceptron (MLP) architecture, renowned for its superior nonlinear function approximation capabilities. For each observed output, a separate ANN model was developed, including yield, TPC, TFC, antioxidant assays (DPPH, ABTS, CUPRAC, and FRAP), and enzyme inhibition activities (α-amylase and tyrosinase). Prior to model construction, both the input and output datasets were normalized to optimize computational efficiency and predictive stability. To ensure the robustness of the models despite the limited sample size (*N* = 27), the data were partitioned into training (60%), testing (20%), and validation (20%) subsets with 100 000 runs to determine the optimal configuration based on the highest predictive performance and minimal error rates.

The internal architecture of the ANN is characterized by weight matrices and bias vectors, where *W*_1_ and *B*_1_ correspond to the hidden layer, and *W*_2_ and *B*_2_ correspond to the output layer, respectively.

The mathematical relationship between the input variables and the predicted responses is defined as follows:

1 *Y* = *f*_1_(*W*_2_·*f*_2_(*W*_1_·*X* + *B*_1_) + *B*_2_)

In this equation, *Y* represents the output matrix, *X* is the matrix of input parameters, and *f*_1_ and *f*_2_ denote the transfer functions for the hidden and output layers, respectively.^26^ During the learning cycle, the weight coefficients *W*_1_ and *W*_2_ were iteratively refined to minimize the residual error between the experimental and predicted values, thereby maximizing the model's reliability.^27^

Nine ANN models were developed for prediction and optimization of the parameters: yield, TPC, TFC, DPPH, ABTS, CUPRAC, FRAP, α-amylase and tyrosinase, according to: solvent concentration, time, temperature and plant to solvent ratio.

### 2.5. Model validation

The predictive accuracy and reliability of the developed ANN models were validated using statistical performance indices, including the coefficient of determination (*r*^2^), reduced chi-square (*χ*^2^), mean bias error (MBE), root mean square error (RMSE), and mean percentage error (MPE), through the application of the following equations. These parameters were derived using the following mathematical equations:^28^2
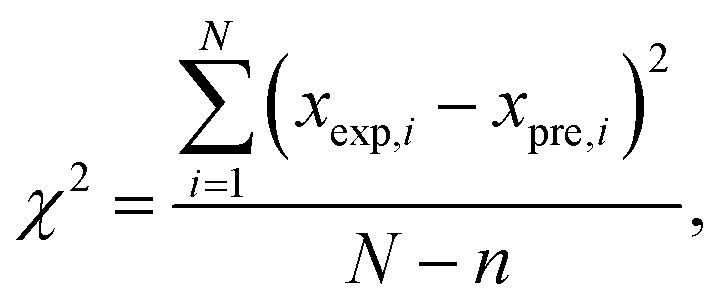
3
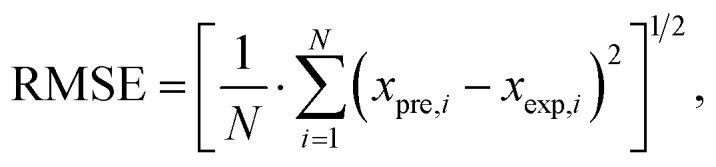
4
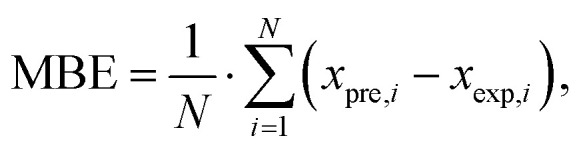
5
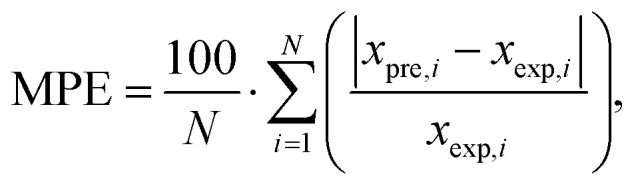
where *x*_exp,*i*_ marks the experimental values and *x*_pre,*i*_ present value obtained by the models, *N* and *n* are the number of observations and constants, respectively (Table 1).

**Table 1:** Extraction yield of the *C. monogyna* extracts

	Conc.	Time	Temp.	Ratio	Yield %
1	50	15	45	30	21.26 ± 0.166^e^
2	90	15	45	30	18.00 ± 0.136^b^
3	50	45	45	30	23.42 ± 0.150^g,h^
4	90	45	45	30	20.41 ± 0.178^c,d^
5	50	30	30	30	21.93 ± 0.172^f^
6	90	30	30	30	17.63 ± 0.181^b^
7	50	30	60	30	25.38 ± 0.209^k,l,m^
8	90	30	60	30	23.75 ± 0.200^h^
9	70	15	30	30	23.62 ± 0.042^h^
10	70	45	30	30	25.30 ± 0.136^k,l^
11	70	15	60	30	25.12 ± 0.301^j,k^
12	70	45	60	30	27.77 ± 0.226^n^
13	50	30	45	15	17.09 ± 0.261^b^
14	90	30	45	15	13.21 ± 0.071^a^
15	50	30	45	45	24.63 ± 0.204^ı,j^
16	90	30	45	45	21.85 ± 0.207^e,f^
17	70	15	45	15	19.98 ± 0.179^c,d^
18	70	45	45	15	22.85 ± 0.313^g^
19	70	15	45	45	24.46 ± 0.123^i^
20	70	45	45	45	29.07 ± 0.190^o^
21	70	30	30	15	19.87 ± 0.162^c^
22	70	30	60	15	20.51 ± 0.139^d^
23	70	30	30	45	25.81 ± 0.226^l,m^
24	70	30	60	45	29.23 ± 0.253^o^
25	70	30	45	30	24.51 ± 0.170^i^
26	70	30	45	30	25.46 ± 0.086^k,l,m^
27	70	30	45	30	25.93 ± 0.085^m^

## 3. Results and discussion

### 3.1. Total bioactive compounds

The total phenolic content (TPC) of *C. monogyna* extracts showed moderate variation across the tested conditions, ranging from 94.42 to 113.04 mg GAE per g extract (Table 2). The highest TPC was observed in sample 1 (113.04 mg GAE per g), followed closely by samples 7 and 13, while the lowest value was recorded in sample 10 (94.42 mg GAE per g).

**Table 2:** Total bioactive compounds and antioxidant capacity of *C. monogyna* extracts^a^

Assays	Total phenolic (mg GAE per g)	Total flavonoid (mg RE per g)	DPPH (mg TE per g)	ABTS (mg TE per g)	CUPRAC (mg TE per g)	FRAP (mg TE per g)
1	113.04 ± 1.78^j^	43.33 ± 1.33^a^	476.06 ± 1.78^l^	548.62 ± 2.24^o^	392.46 ± 2.59^j,k^	379.77 ± 1.12^j,k^
2	97.98 ± 1.37^a,b,c^	67.91 ± 0.72^d,e,f,g,h^	439.65 ± 0.76^b,c,d,e,f,g^	482.59 ± 2.24^b,c,d^	350.70 ± 0.34^a,b,c,d^	341.01 ± 2.35^b^
3	104.85 ± 0.97^f,g,h,i^	52.66 ± 0.09^a,b,c,d,e^	455.36 ± 0.67^g,h,i,j,k^	511.46 ± 9.95^g,h,i,j,k,l^	349.91 ± 10.99^a,b,c,d^	360.51 ± 2.22^e,f,g^
4	101.21 ± 0.66^c,d,e,f^	72.16 ± 2.00^e,f,g,h^	447.72 ± 1.27^d,e,f,g,h,i^	496.39 ± 6.99^c,d,e,f,g,h^	367.13 ± 1.24^c,d,e,f,g,h,i^	364.29 ± 3.28^g,h^
5	101.21 ± 0.96^c,d,e,f,g^	54.70 ± 0.61^a,b,c,d,e,f^	451.10 ± 3.84^e,f,g,h,i,j^	503.18 ± 11.90^e,f,g,h,i,j,k^	375.24 ± 3.09^e,f,g,h,i,j^	372.33 ± 1.69^h,i,j,k^
6	104.33 ± 2.35^e,f,g,h^	78.79 ± 0.29^h^	468.87 ± 0.25^k,l^	514.65 ± 1.10^h,i,j,k,l^	387.31 ± 4.84^i,j,k^	392.44 ± 2.35^l^
7	112.73 ± 0.86^j^	59.19 ± 0.69^a,b,c,d,e,f,g,h^	468.43 ± 0.92^k,l^	546.50 ± 4.18^o^	386.32 ± 9.65^i,j,k^	391.22 ± 1.83^l^
8	95.44 ± 0.78^a,b^	67.69 ± 1.02^d,e,f,g,h^	421.88 ± 10.20^a^	457.32 ± 2.92^a^	334.47 ± 1.24^a,b^	323.71 ± 2.20^a^
9	105.80 ± 0.54^g,h,i^	71.30 ± 1.23^e,f,g,h^	466.96 ± 3.44^j,k,l^	509.55 ± 1.10^f,g,h,i,j,k,l^	384.94 ± 9.76^h,i,j,k^	374.53 ± 2.59^j,k^
10	94.42 ± 0.65^a^	54.05 ± 8.26^a,b,c,d,e,f^	454.33 ± 4.59^f,g,h,i,j,k^	490.87 ± 2.87^c,d,e,f^	352.48 ± 11.95^a,b,c,d,e^	351.49 ± 4.21^c,d,e^
11	103.14 ± 1.28^d,e,f,g^	64.67 ± 0.24^c,d,e,f,g,h^	465.05 ± 5.61^j,k,l^	516.99 ± 4.24^i,j,k,l,m^	365.94 ± 7.33^c,d,e,f,g,h,i^	381.23 ± 3.19^k^
12	100.38 ± 0.16^c,d,e^	51.06 ± 10.75^a,b,c,d,e^	441.85 ± 3.44^c,d,e,f,g,h^	520.81 ± 2.24^k,l,m,n^	362.38 ± 0.69^c,d,e,f,g,h^	364.53 ± 4.22^g,h,i^
13	112.47 ± 0.66^j^	54.13 ± 10.03^a,b,c,d,e,f^	467.11 ± 3.09^j,k,l^	522.93 ± 1.10^l,m,n^	388.11 ± 2.09^i,j,k^	397.44 ± 5.88^l^
14	105.82 ± 1.36^g,h,i^	72.72 ± 0.89^f,g,h^	465.35 ± 2.93^j,k,l^	513.16 ± 2.87^g,h,i,j,k,l^	380.39 ± 9.20^g,h,i,j^	373.31 ± 0.73^h,i,j,k^
15	104.09 ± 0.81^e,f,g,h^	45.90 ± 10.03^a,b,c^	424.67 ± 7.09^a,b^	484.29 ± 20.32^b,c,d^	376.03 ± 6.75^f,g,h,i,j^	373.67 ± 2.64^i,j,k^
16	101.43 ± 0.85^c,d,e,f,g^	75.98 ± 1.58^g,h^	438.77 ± 1.59^b,c,d,e,f^	466.03 ± 0.74^a,b^	357.43 ± 7.22^c,d,e,f^	346.86 ± 1.48^b,c,d^
17	94.92 ± 1.17^a,b^	63.55 ± 0.28^b,c,d,e,f,g,h^	439.65 ± 3.44^b,c,d,e,f,g^	479.19 ± 4.82^b,c^	334.08 ± 7.75^a^	345.76 ± 0.21^b,c,d^
18	103.95 ± 1.04^e,f,g,h^	50.84 ± 9.82^a,b,c,d^	435.98 ± 7.27^a,b,c,d,e^	509.13 ± 3.51^f,g,h,i,j,k,l^	371.48 ± 6.09^d,e,f,g,h,i,j^	370.75 ± 1.27^h,i,j^
19	102.07 ± 2.56^c,d,e,f,g^	63.92 ± 3.44^b,c,d,e,f,g,h^	429.37 ± 13.16^a,b,c^	489.17 ± 1.27^c,d,e^	353.27 ± 10.35^a,b,c,d,e,f^	359.54 ± 1.28^e,f,g^
20	101.52 ± 0.30^c,d,e,f,g^	44.40 ± 4.38^a,b^	425.40 ± 6.36^a,b^	500.42 ± 0.97^d,e,f,g,h,i,j^	348.52 ± 4.71^a,b,c^	364.05 ± 4.83^g,h^
21	100.60 ± 0.47^c,d,e,f^	66.36 ± 0.91^d,e,f,g,h^	460.06 ± 7.70^i,j,k,l^	500.64 ± 6.89^d,e,f,g,h,i,j^	362.38 ± 3.43^c,d,e,f,g,h^	361.00 ± 3.88^f,g^
22	108.01 ± 3.60^h,i^	48.81 ± 12.77^a,b,c,d^	432.89 ± 7.16^a,b,c,d^	535.03 ± 4.59^m,n,o^	390.08 ± 4.16^j,k^	392.20 ± 2.43^l^
23	99.91 ± 0.45^c,d,e^	57.18 ± 9.80^a,b,c,d,e,f,g^	456.39 ± 2.75^h,i,j,k^	479.19 ± 0.74^b,c^	352.28 ± 9.80^a,b,c,d^	352.71 ± 2.01^d,e,f^
24	102.24 ± 0.33^d,e,f,g^	45.07 ± 14.74^a,b,c^	443.91 ± 1.35^c,d,e,f,g,h^	498.51 ± 3.27^d,e,f,g,h,i^	360.40 ± 5.73^c,d,e,f,g^	364.90 ± 1.10^g,h,i^
25	109.15 ± 2.88^ı,j^	70.74 ± 1.18^e,f,g,h^	473.57 ± 0.44l	537.58 ± 3.87^n,o^	407.11 ± 15.65^k^	393.91 ± 1.88^l^
26	99.34 ± 0.93^b,c,d^	61.06 ± 1.12^a,b,c,d,e,f,g,h^	451.84 ± 1.67^e,f,g,h,i,j^	518.05 ± 0.74^j,k,l,m^	352.88 ± 2.81^a,b,c,d,e^	342.72 ± 3.73^b,c^
27	100.19 ± 1.79^c,d,e^	62.69 ± 0.30^a,b,c,d,e,f,g,h^	434.51 ± 2.58^a,b,c,d^	494.48 ± 0.37^c,d,e,f,g^	357.23 ± 2.40^b,c,d,e,f^	364.29 ± 4.42^g,h^

a Values expressed are means ± S.D. GAE: gallic acid equivalents. RE: rutin equivalents. TE: Trolox equivalent.

Overall, the relatively narrow range of TPC values indicates that all applied ultrasound-assisted extraction (UAE) conditions were effective for phenolic extraction, with only a moderate influence of process parameters. Slightly higher TPC values were generally associated with lower ethanol concentrations and moderate-to-elevated temperatures, suggesting that these conditions may favor the extraction of a broader spectrum of phenolic compounds. The absence of pronounced differences between samples implies that phenolic extraction is governed by the combined effects of multiple parameters rather than a single dominant factor.

The total phenolic content obtained in this study was lower than the values reported by Ülger *et al.* (2023),^29^ which ranged from 116.24 to 301.77 mg GAE per g. These differences may be attributed to variations in extraction conditions, including solvent composition and process parameters. It has been widely reported that phenolic and flavonoid content is significantly influenced by factors such as extraction methodology, geographical origin, genotype, and growth stage of the plant material.^30–32^

In contrast, the TPC values obtained in this study were higher than those reported for *C. monogyna* fruits by Parzhanova *et al.* (2023),^33^ suggesting that flowering branches may represent a richer source of phenolic compounds. This observation is in line with previous reports identifying *C. monogyna* as a source of various bioactive phenolics, including catechin, chlorogenic acid, and quercetin.^21,34^

The total flavonoid content (TFC) exhibited a wider variation compared to TPC, ranging from 43.33 to 78.79 mg RE per g extract. The highest TFC was obtained in sample 6 (78.79 mg RE per g), while the lowest value was observed in sample 1 (43.33 mg RE per g), which corresponded to the highest TPC.

This inverse tendency observed between TPC and TFC in certain samples suggests that non-flavonoid phenolics contribute substantially to the total phenolic content, and that extraction conditions may selectively favor different subclasses of phenolic compounds. In particular, higher ethanol concentrations appear to promote the extraction of flavonoids, while lower concentrations may favor a broader range of phenolic constituents.

From a mechanistic perspective, this behavior can be attributed to ultrasound-assisted extraction (UAE)-induced cell wall disruption and cavitation effects, which enhance mass transfer but also differentially release intracellular phenolic fractions depending on their polarity and binding forms within plant tissues.

Similar trends have been reported in previous studies on different plants,^35,36^ where solvent polarity was identified as a key factor influencing the selectivity of phenolic subclasses during extraction. These findings confirm that TPC alone does not fully represent extract composition, but rather reflects the combined contribution of structurally diverse phenolic groups.

The greater variability of TFC compared to TPC further indicates that flavonoids are more sensitive to changes in extraction parameters. This behavior may be associated with their structural diversity, differential solubility, and potential susceptibility to degradation under ultrasonic conditions. From a practical standpoint, these results highlight the possibility of tailoring extraction conditions to obtain extracts enriched either in total phenolics or specifically in flavonoid fractions, depending on the desired biological application.^37–39^

### 3.2. Biological properties (antioxidant capacity and enzyme inhibition)

The antioxidant activity of *C. monogyna* extracts, evaluated using DPPH, ABTS, CUPRAC, and FRAP assays, showed moderate variability depending on the extraction conditions (Table 2). DPPH values ranged from 421.88 to 476.06 mg TE per g extract, with the highest activity observed in sample 1, followed by samples 25, 6, and 7. The lowest activity was recorded in sample 8.

A similar trend was observed for ABTS, with values ranging from 457.32 to 548.62 mg TE per g extract, where sample 1 and sample 7 exhibited the highest activity, while sample 8 again showed the lowest value. CUPRAC and FRAP assays followed comparable patterns, with the highest reducing capacities observed in samples 25 and 13, while the lowest values were recorded in samples 8 and 17.

The consistency across different antioxidant assays suggests that the observed variations are not assay-specific, but rather reflect real differences in the chemical composition of the extracts. Notably, samples exhibiting higher antioxidant activity generally corresponded to those with higher total phenolic content, confirming the significant contribution of phenolic compounds to antioxidant potential.

However, this relationship was not strictly linear, indicating that antioxidant activity depends not only on the total amount of phenolics, but also on their composition and structural characteristics. Different phenolic subclasses possess varying redox properties, which can explain the differences observed between samples with similar TPC values.

The relatively moderate variation in antioxidant activity across samples further supports the robustness of UAE as an extraction technique, providing efficient recovery of antioxidant compounds under a wide range of conditions. At the same time, slightly enhanced activities observed under specific conditions suggest that optimization of extraction parameters can still improve antioxidant performance.

Overall, the results indicate that *C. monogyna* extracts exhibit consistently high antioxidant potential, primarily driven by phenolic compounds, while subtle variations in extraction conditions allow for fine-tuning of antioxidant efficiency.

Our findings align with previous studies demonstrating that optimized UAE conditions significantly improve the recovery of phenolic compounds from various *Crataegus* species.^21,40,41^ Previous study have shown that UAE conditions (the extraction time, percentage of acetone, and solvent-to-solid ratio) could effect of bioactive compounds and antioxidant properties (DPPH, ABTS, FRAP) from *Crataegus monogyna* leaves.^21^*C. monogyna* leaf/flower and berries have been reported to optimize extraction conditions for radical scavenging activity (DPPH) in a previous study.^23^ A different study highlighted that the extraction conditions can influence the recovery of phenolic compounds from *C. laciniata* leaves.

The enzyme inhibitory activities of *C. monogyna* extracts against α-amylase and tyrosinase are presented in Table 3. The α-amylase inhibitory activity showed relatively low variability, ranging from 0.45 to 0.66 mmol ACAE per g extract. The highest inhibition was observed in samples 2 and 4, while the lowest activity was recorded in samples 21 and 27.

**Table 3:** Enzyme inhibition of the *C. monogyna* extracts^a^

Assays	α-amylase inhibition (mmol ACAE per g extract)	Tyrosinase inhibition (mg KAE per g extract)
1	0.50 ± 0.02^a^	74.12 ± 0.64^a^
2	0.66 ± 0.04^a,b^	75.26 ± 2.27^a,b^
3	0.54 ± 0.02^a,b,c^	74.48 ± 0.32^a,b,c^
4	0.66 ± 0.05^a,b,c^	76.25 ± 0.16^a,b,c,d^
5	0.53 ± 0.04^a,b,c^	74.20 ± 0.04^a,b,c,d^
6	0.57 ± 0.01^a,b,c,d^	77.41 ± 0.70^a,b,c,d^
7	0.52 ± 0.03^a,b,c,d^	74.80 ± 0.50^a,b,c,d,e^
8	0.59 ± 0.03^a,b,c,d^	77.16 ± 0.34^a,b,c,d,e,f^
9	0.52 ± 0.01^a,b,c,d^	77.75 ± 0.20^a,b,c,d,e,f^
10	0.52 ± 0.02^a,b,c,d,e^	76.56 ± 0.23^b,c,d,e,f,g^
11	0.50 ± 0.04^a,b,c,d,e^	76.51 ± 0.42^b,c,d,e,f,g,h^
12	0.53 ± 0.01^a,b,c,d,e^	76.47 ± 0.32^b,c,d,e,f,g,h^
13	0.52 ± 0.02^a,b,c,d,e^	75.48 ± 0.17^b,c,d,e,f,g,h,i^
14	0.64 ± 0.03^a,b,c,d,e^	75.38 ± 0.84^b,c,d,e,f,g,h,i^
15	0.53 ± 0.03^b,c,d,e^	76.84 ± 0.19^c,d,e,f,g,h,i,j^
16	0.53 ± 0.06^b,c,d,e^	76.63 ± 0.28^d,e,f,g,h,i,j^
17	0.50 ± 0.05^c,d,e^	75.75 ± 0.72^e,f,g,h,i,j^
18	0.55 ± 0.04^c,d,e^	74.55 ± 0.15^e,f,g,h,i,j^
19	0.57 ± 0.02^c,d,e^	76.56 ± 0.26^e,f,g,h,i,j^
20	0.60 ± 0.02^c,d,e,f^	75.79 ± 0.13^e,f,g,h,i,j^
21	0.45 ± 0.03^c,d,e,f^	75.99 ± 0.12^e,f,g,h,i,j^
22	0.51 ± 0.01^d,e,f,g^	76.95 ± 0.17^f,g,h,i,j^
23	0.54 ± 0.01^d,e,f,g^	76.38 ± 0.10^f,g,h,i,j^
24	0.58 ± 0.03^f,g^	74.45 ± 0.81^g,h,i,j^
25	0.53 ± 0.01^f,g^	75.00 ± 0.21^h,i,j^
26	0.55 ± 0.01^g^	74.88 ± 0.17^i,j^
27	0.46 ± 0.02^g^	73.30 ± 0.23^j^

a Values expressed are means ± SD. ACE: acarbose equivalents; KAE: kojic acid equivalents.

Similarly, tyrosinase inhibition exhibited a narrow range of values, from 73.30 to 77.75 mg KAE per g extract, with the highest activity observed in sample 9, followed by samples 6 and 8, while the lowest value was recorded in sample 27.

The limited variability in enzyme inhibition across different extraction conditions suggests that the bioactive compounds responsible for these activities are efficiently extracted under all applied UAE conditions. This indicates that, unlike flavonoids, enzyme inhibitory activity is less sensitive to variations in extraction parameters within the tested range.

Although phenolic compounds are known to contribute to enzyme inhibition, no clear direct correlation between total phenolic content and enzyme inhibitory activity was observed. This suggests that specific compounds or subclasses of phenolics, rather than total phenolic content, play a dominant role in enzyme inhibition.

The observed α-amylase inhibitory activity highlights the potential of *C. monogyna* extracts for applications related to glycemic control, while tyrosinase inhibition suggests possible use in cosmetic formulations and skin-related applications. The relatively stable inhibition profiles across samples indicate that bioactivity can be reliably obtained without the need for highly restrictive extraction conditions.

Overall, the results demonstrate that *C. monogyna* extracts possess consistent enzyme inhibitory potential, with limited sensitivity to extraction parameters, suggesting that the key bioactive constituents responsible for these effects are readily extractable under a broad range of UAE conditions.

### 3.3. Correlation analysis

**Fig. 1:**
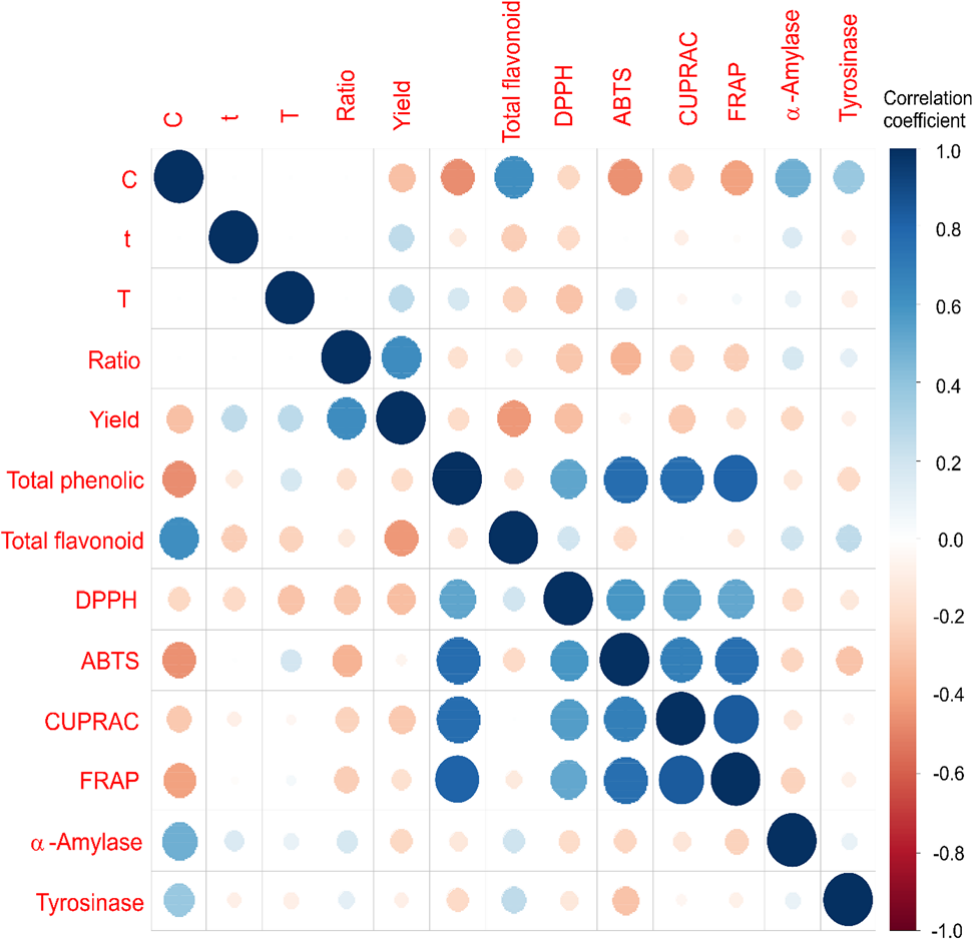
The correlation analysis between observed responses of *C. monogyna* flowering branches extract samples.

The correlation analysis revealed that total phenolic content was the main determinant of antioxidant capacity in *Crataegus monogyna* extracts, Fig. 1. Strong positive correlations were observed between total phenolics and FRAP (*r* = 0.808), ABTS (*r* = 0.763), CUPRAC (*r* = 0.762), and DPPH (*r* = 0.529) (*p* < 0.001), indicating that phenolic compounds predominantly contributed to the reducing power and radical scavenging activities. Antioxidant assays were also strongly intercorrelated, particularly FRAP and CUPRAC (*r* = 0.837), confirming methodological consistency and similar electron-transfer mechanisms. In contrast, extraction yield showed significant negative correlations with total flavonoids (*r* = −0.436, *p* < 0.001), DPPH (*r* = −0.304, *p* = 0.006), and CUPRAC (*r* = −0.269, *p* = 0.015), suggesting that higher mass recovery did not correspond to higher bioactive potency and may reflect co-extraction of non-phenolic constituents. Total flavonoids were positively associated with tyrosinase inhibition (*r* = 0.256, *p* = 0.021), whereas enzyme inhibitory activities showed generally weak or nonsignificant correlations with antioxidant parameters, indicating that these biological effects are likely governed by specific compounds rather than total phenolic concentration alone. Overall, the results indicate that phenolic content is the primary contributor to antioxidant activity, whereas enzyme inhibition involves more selective phytochemical interactions.

### 3.4. Cluster analysis

**Fig. 2:**
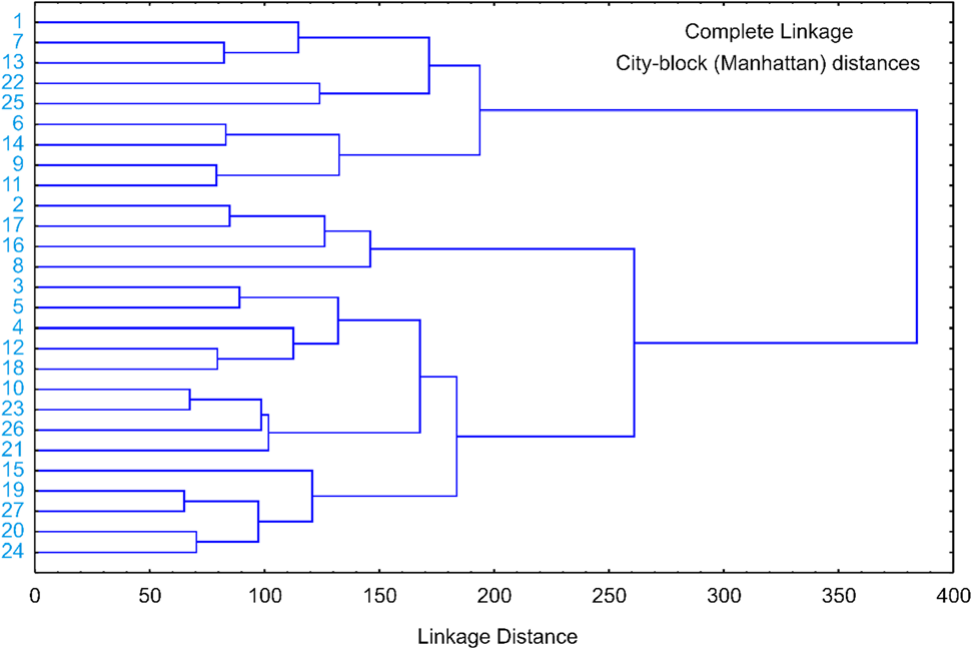
Cluster of *C. monogyna* flowering branch extract samples based on observed responses.

Hierarchical cluster analysis was performed using complete linkage and city-block (Manhattan) distances to objectively group the 27 extracts based on their multi-parameter profiles, revealing two primary clusters that underscore the impact of extraction conditions on bioactive composition (Fig. 2).

The first major cluster is characterized by samples with high antioxidant potency, notably samples 1, 7, and 13 (all 50% ethanol), which exhibit the shortest linkage distances due to their consistently high phenolic content and superior ABTS values. The second major cluster encompasses extracts with higher yields and specialized enzymatic inhibition; within this group, samples 20 and 24 are tightly linked, reflecting their shared characteristics as the highest-yielding extracts in the study. Furthermore, the distinct sub-branching of 90% ethanol extracts, as seen in samples 2, 4, and 14, confirms that solvent concentration is a decisive factor in differentiating extracts, effectively separating those with high enzyme inhibitory potential from those with high overall yields. The high degree of similarity observed between replicates 25, 26, and 27 at the center of the dendrogram statistically validates the reproducibility of the extraction process under intermediate conditions.

### 3.5. Principal component analysis (PCA)

**Fig. 3:**
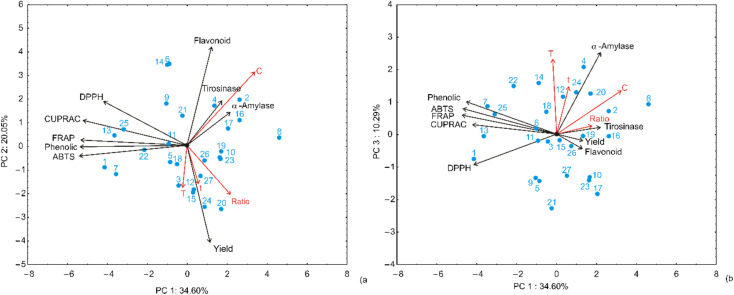
Principal component analysis (PCA) of *C. monogyna* flowering branch extract samples based on observed responses.

The multivariate assessment of the 27 extract samples using Principal Component Analysis (PCA) revealed three principal components explaining 64.97% of the total variance (Fig. 3), effectively elucidating the correlation between extraction parameters-ethanol concentration, time, temperature, and solvent-to-solid ratio- and their resulting chemical profiles. PC1 accounted for 34.60% of the variance, primarily discriminating samples based on antioxidant density, where extracts obtained with 50% ethanol (*e.g.*, samples 1, 7, and 13) exhibit the highest phenolic content (up to 113.04 mg GAE per g) and superior antioxidant capacities (ABTS and CUPRAC), clustering on the far left of the biplot. PC2 explained 20.05% of the variance and further discriminated samples based on extraction time and solvent-to-solid ratio. Longer extraction times and higher ratios tended to shift samples along the positive direction of PC2, where a stronger association with enzyme inhibition activities (α-amylase and tyrosinase) was observed. The orientation of the loading vectors indicates moderate to strong correlations among antioxidant assays. At the same time, enzyme inhibition activities showed a partially independent pattern, suggesting that different groups of compounds may be responsible for these.

### 3.6. Artificial neural networks

Artificial Neural Networks (ANNs) were developed to model the observed responses, including yield, TPC, TFC, antioxidant assays (DPPH, ABTS, CUPRAC, and FRAP), and enzyme inhibition activities (α-amylase and tyrosinase). Nine separate ANN models were constructed (ANN1–ANN9), with their architectures and parameters presented in Tables S1–S28.

Developing separate ANN models for each response variable improved predictive performance, as each network could capture the specific patterns, data ranges, and variability characteristic of each response.^42^ This strategy allowed the model architecture, hidden-layer configuration, and weight optimization to be tailored to each output, avoiding the potential loss of accuracy that may arise when fitting multiple responses simultaneously within a single network. The developed models are presented in Tables S1, S4, S7, S10, S13, S16, S19, S22, and S25, demonstrating good generalization ability and reliable prediction of the observed parameters. The corresponding weight coefficients and biases for the input layers are provided in Tables S2, S5, S8, S11, S14, S17, S20, S23, and S26, while those for the output layers are shown in Tables S3, S6, S9, S12, S15, S28, S21, S24, and S27.

According to the ANN optimization results, the optimal numbers of neurons in the hidden layer were 5, 6, 8, 7, 6, 10, 4, 8, and 6, corresponding to the MLP 4–5–1, MLP 4–6–1, MLP 4–8–1, MLP 4–7–1, MLP 4–6–1, MLP 4–10–1, MLP 4–4–1, MLP 4–8–1, and MLP 4–6–1 architectures, respectively. During the training phase, the models achieved high coefficients of determination (*r*^2^), ranging from 0.813 to 0.996.

Although slightly lower *r*^2^ values were observed for some models (*e.g.*, ANN7), these results may reflect greater experimental variability, a narrower response range, or a more complex, less linear relationship between input factors and the specific output variable. Nevertheless, even these models demonstrated acceptable predictive performance, indicating that the ANN approach adequately captured the underlying trends in the data. Overall, the obtained *r*^2^ values confirm the robustness, reliability, and strong predictive capability of the developed ANN models.

**Fig. 4:**
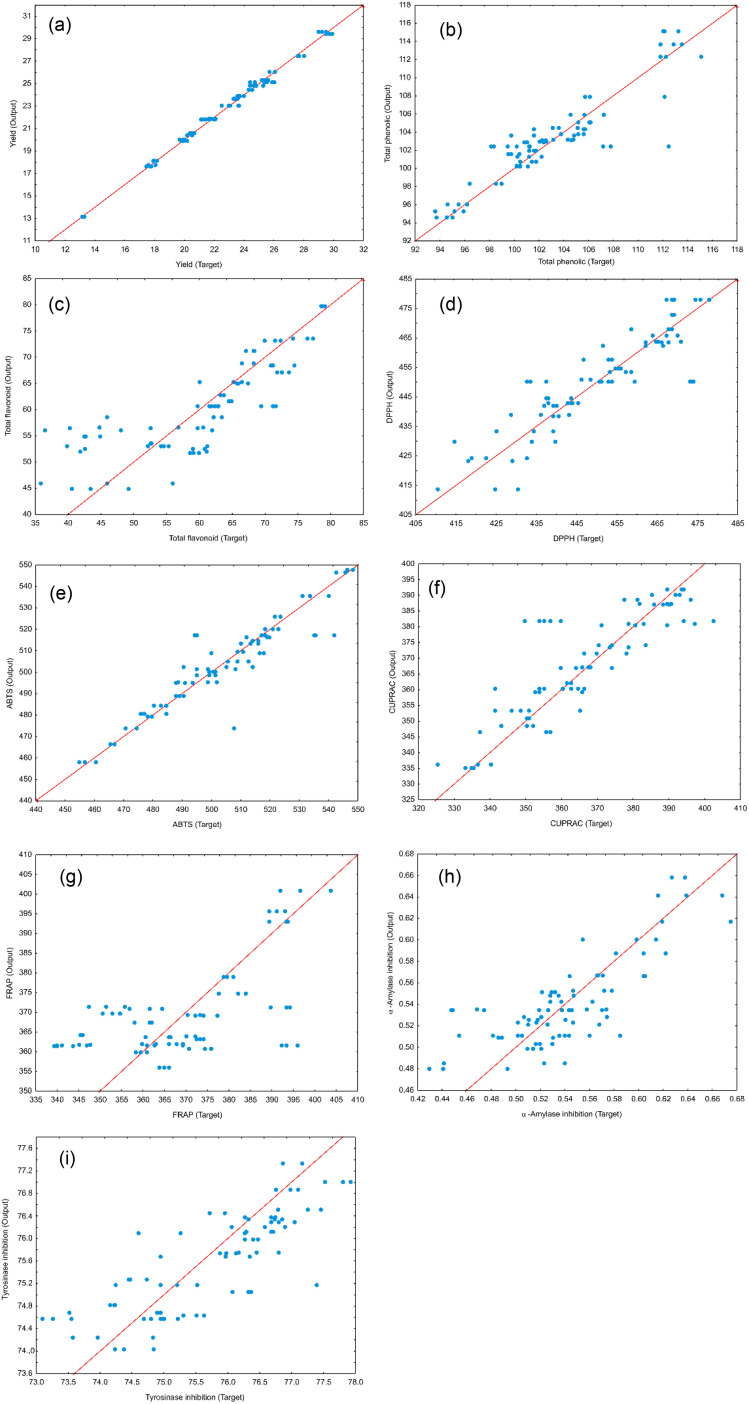
Experimentally obtained and ANN models delivered value comparison for: (a) yield, (b) total phenolic, (c) total flavonoid, (d) DPPH, (e) ABTS, (f) CUPRAC, (g) FRAP, (h) α-amylase inhibition, and (i) tyrosinase inhibition.

The performance of the proposed ANN models was assessed using several metrics, including reduced chi-square (*χ*^2^), root mean square error (RMSE), mean bias error (MBE), mean percentage error (MPE), and the coefficient of determination (*r*^2^) (Table S28). The results in Table S28 demonstrate a high degree of predictive accuracy across most studied parameters, particularly for yield (*r*^2^ = 0.994) and ABTS (*r*^2^ = 0.972). The exceptionally low values for reduced *χ*^2^ and RMSE for the yield model indicate a near-perfect alignment between experimental and predicted data. Similarly, high coefficients of determination (*r*^2^ > 0.900) for TPC, DPPH, and CUPRAC suggest that the neural networks effectively captured the non-linear relationships inherent in these antioxidant assays. However, the model performance for FRAP (*r*^2^ = 0.558) and tyrosinase (*r*^2^ = 0.694) was notably lower, characterized by higher RMSE and *χ*^2^ values, suggesting that these specific bioactivities may be influenced by complex synergistic effects or experimental noise that the current network architecture found challenging to map. Overall, the negligible MBE across all variables confirms that the models did not suffer from significant systematic overestimation or underestimation, validating the ANN as a robust tool for the optimization and prediction of most analysed output parameters (Fig. 4).

### 3.7. ANN optimization and standard score analysis

**Fig. 5:**
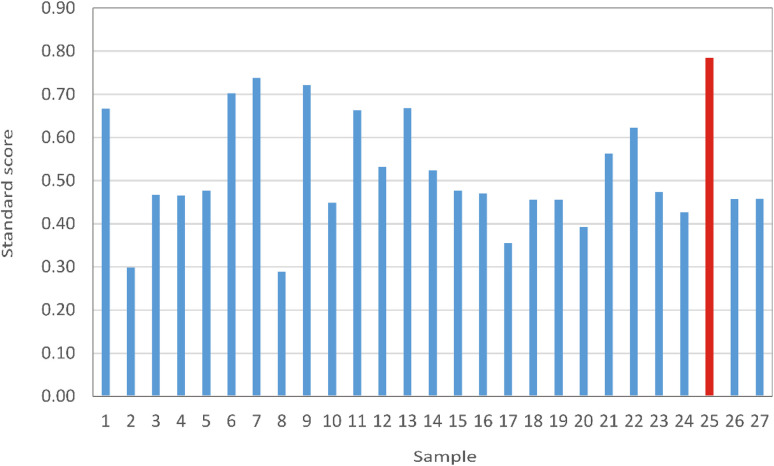
*Z*-score based evaluation of samples 1–27.

The optimization of the ANN outputs was conducted based on the data presented in Tables S1–S27, applying eqn (1). The objective was to maximize the extraction yield, total phenolic content, total flavonoid content, antioxidant activities (DPPH, ABTS, CUPRAC, and FRAP), and enzyme inhibitory activities (α-amylase and tyrosinase) of the *C. monogyna* flowering branch extract. The ANN models included solvent concentration, extraction time, temperature, and solid-to-liquid ratio as input variables. Optimization was performed using eight models (ANN1–ANN8), with 69–94 generations. A population size of 100 was applied for each input variable, and 50 solutions were selected along the Pareto front. All ANN models identified sample 25 as the optimal extract. The optimization results were further validated using standard score (*Z*-score) analysis, in which the mean *Z*-score across all evaluated responses was calculated according to the well-known methodology.^43^ Sample 25 exhibited the highest overall *Z*-score (0.78), confirming it as the optimal extraction condition (Fig. 5). This extract represents the optimal balance between antioxidant capacity and enzyme-inhibitory potential, demonstrating the robustness and reliability of the ANN-based optimization approach for defining optimal extraction parameters.

The ANN model optimization and standard score analysis identified sample 25 as the optimal extract. This sample was obtained using solvent concentration 70%, 30 min UAE at 45 °C, with a sample to solvent ratio of 1 : 30, with yield of 24.51%.

The measured values for this optimal *C. monogyna* flowering branches extract samples were as follows:

- Total phenolics: 109.15 ± 2.88 mg GAE per g

- Total flavonoids: 70.74 ± 1.18 mg RE per g

-Antioxidant assays:

DPPH: 473.57 ± 0.44 mg TE per g,

ABTS: 537.58 ± 3.87 mg TE per g,

CUPRAC: 407.11 ± 15.65 mg TE per g,

FRAP: 393.91 ± 1.88 mg TE per g,

- Enzyme inhibition assays:

α-amylase inhibition 0.53 ± 0.01 mmol ACE per g extract

Tyrosinase inhibition 75.00 ± 0.21 mg KAE per g extract

## 4. Conclusions

The present study demonstrated that extraction parameters, particularly solvent concentration, temperature, and plant-to-solvent ratio, play a critical role in determining both the chemical composition and biological activity of *C. monogyna* extracts. Specifically, extracts obtained with 50% ethanol exhibited the highest total phenolic content and antioxidant capacity confirming the key contribution of phenolic compounds to antioxidant performance.

In contrast, enzyme inhibitory activities showed a more selective behavior, suggesting the involvement of specific bioactive constituents rather than total phenolic levels alone.

The developed ANN models (MLP 4–5–1 for yield and MLP 4–6–1 for TPC) exhibited high predictive accuracy, confirming their suitability for modeling complex, nonlinear extraction processes and enabling reliable optimization. The optimal extraction conditions (70% ethanol, 30 min, 45 °C, 1 : 30 ratio) resulted in extracts with a well-balanced profile of bioactive compounds and biological activities. Overall, this study provides a robust framework for the rational optimization of extraction processes and highlights the potential of *C. monogyna* flowering branches as a valuable source of bioactive compounds for future pharmaceutical and functional applications.

## Conflicts of interest

All the authors declare that there is no conflicts of interest.

## Supplementary Material

RA-016-D6RA03013K-s001

## Data Availability

The data supporting the findings of this study are available from the corresponding author upon reasonable request. Supplementary information (SI): details for biological activities and optimization. See DOI: https://doi.org/10.1039/d6ra03013k.
