# Supplementary material for: Optimization of ultrasound-assisted extraction and biological activities of Crataegus monogyna Jacq. flowering branches using experimental design and artificial neural networks
Source: RSC Adv. 2026 May 26;16(31):28573–82. doi: 10.1039/d6ra03013k (PMC13213556; doi:10.1039/d6ra03013k)
Supplement: RA-016-D6RA03013K-s001 [file RA-016-D6RA03013K-s001.pdf]

Supplementary Information (SI) for RSC Advances.
This journal is © The Royal Society of Chemistry 2026

Optimization of ultrasound-assisted extraction and biological activities of *Crataegus monogyna* Jacq. flowering branches using experimental design and artificial neural networks

Sengul Uysal[1,2*], Aleksandra Cvetanović Kljakić[3], Biljana Lončar[3], Gokhan Zengin[4], Ugur Cakilcioglu[5]

[1]Erciyes University, Halil Bayraktar Health Services Vocational College, Kayseri, Türkiye

[2]Erciyes University, Drug Application and Research Center, 38280, Kayseri, Türkiye

[3]Faculty of Technology Novi Sad, University of Novi Sad, 21000, Novi Sad, Serbia

[4]Selcuk University, Department of Biology, Science Faculty, Konya, Turkey

[5]Munzur University, Pertek Sakine Genç Vocational School, Pertek, Tunceli, Turkey

*Corresponding author: Dr. Sengul UYSAL (senguluysal@erciyes.edu.tr)

Total phenolic and flavonoid content

The total phenolic content was determined by employing the Folin-Ciocalteu method. Sample solution (1 mg/mL; 0.25 mL) was mixed with diluted Folin–Ciocalteu reagent (1 mL, 1:9, v/v) and shaken vigorously. After 3 min, Na_2CO_3 solution (0.75 mL, 1%) was added and the sample absorbance was read at 760 nm after a 2 h incubation at room temperature. The total phenolic content was expressed as milligrams of gallic acid equivalents (mg GAE/g extract)

The total flavonoids content was determined using $AlCl_3$ method. Briefly, sample solution (1 mg/mL;1 mL) was mixed with the same volume of aluminum trichloride (2%) in methanol. Similarly, a blank was prepared by adding sample solution (1 mL) to methanol (1 mL) without $AlCl_3$. The sample and blank absorbances were read at 415 nm after a 10 min incubation at room temperature. The absorbance of the blank was subtracted from that of the sample. Rutin was used as a reference standard and the total flavonoid content was expressed as milligrams of rutin equivalents (mg RE/g extract).

Antioxidant and Enzyme Inhibition assays

Antioxidant (DPPH and ABTS radical scavenging, reducing power (CUPRAC and FRAP)) tyrosinase (dopachrome method), and α-amylase (iodine/potassium iodide method) were determined.

For the DPPH (1,1-diphenyl-2-picrylhydrazyl) radical scavenging assay: Sample solution (1 mg/mL; 1 mL) was added to 4 mL of a 0.004% methanol solution of DPPH. The sample absorbance was read at 517 nm after a 30 min incubation at room temperature in the dark. DPPH radical scavenging activity was expressed as millimoles of trolox equivalents (mg TE/g extract).

For ABTS (2,20 -azino-bis(3-ethylbenzothiazoline) 6-sulfonic acid) radical scavenging assay: Briefly, $ABTS^+$ was produced directly by reacting 7 mM ABTS solution with 2.45 mM potassium persulfate and allowing the mixture to stand for 12–16 in the dark at room temperature. Prior to beginning the assay, ABTS solution was diluted with methanol to an absorbance of 0.700 ± 0.02 at 734 nm. Sample solution (1 mg/mL; 1 mL) was added to ABTS solution (2 mL) and mixed. The sample absorbance was read at 734 nm after a 30 min incubation at room temperature. The ABTS radical scavenging activity was expressed as millimoles of trolox equivalents (mmol TE/g extract).

For CUPRAC (cupric ion reducing activity) activity assay: Sample solution (1 mg/mL; 0.5 mL) was added to premixed reaction mixture containing $CuCl_2$ (1 mL, 10 mM), neocuproine (1 mL, 7.5 mM) and NH4Ac buffer (1 mL, 1 M, pH 7.0). Similarly, a blank was prepared by adding sample solution (0.5 mL) to premixed reaction mixture (3 mL) without $CuCl_2$. Then, the sample and blank absorbances were read at 450 nm after a 30 min incubation at room temperature. The absorbance of the blank was subtracted from that of the sample. CUPRAC activity was expressed as milligrams of trolox equivalents (mg TE/g extract).

For FRAP (ferric reducing antioxidant power) activity assay: Sample solution (1 mg/mL; 0.1 mL) was added to premixed FRAP reagent (2 mL) containing acetate buffer (0.3 M, pH 3.6), 2,4,6-tris(2-pyridyl)-S-triazine (TPTZ) (10 mM) in 40 mM HCl and ferric chloride (20 mM) in a ratio of 10:1:1 (v/v/v). Then, the sample absorbance was read at 593 nm after a 30 min incubation at room temperature. FRAP activity was expressed as milligrams of trolox equivalents (mg TE/g extract).

For Tyrosinase inhibitory activity assay: Sample solution (1 mg/mL; 25 μL) was mixed with tyrosinase solution (40 μL, Sigma) and phosphate buffer (100 μL, pH 6.8) in a 96-well microplate and incubated for 15 min at 25◦C. The reaction was then initiated with the addition of L-DOPA (40 μL, Sigma). Similarly, a blank was prepared by adding sample solution to all reaction reagents without enzyme (tyrosinase) solution. The sample and blank absorbances were read at 492 nm after a 10 min incubation at 25◦C. The absorbance of the blank was subtracted from that of the sample and the tyrosinase inhibitory activity was expressed as kojic acid equivalents (mgKAE/g extract).

For α-amylase inhibitory activity assay: Sample solution (1 mg/mL; 25 μL) was mixed with α-amylase solution (ex-porcine pancreas, EC 3.2.1.1, Sigma) (50 μL) in phosphate buffer (pH 6.9 with 6 mM sodium chloride) in a 96-well microplate and incubated for 10 min at 37◦C. After pre-incubation, the reaction was initiated with the addition of starch solution (50 μL, 0.05%). Similarly, a blank was prepared by adding sample solution to all reaction reagents without enzyme (α-amylase) solution. The reaction mixture was incubated 10 min at 37◦C. The reaction was then stopped with the addition of HCl (25 μL, 1 M). This was followed by addition of the iodine-potassium iodide solution (100 μL). The sample and blank absorbances were read at 630 nm. The absorbance of the blank was subtracted from that of the sample and the α-amylase inhibitory activity was expressed as acarbose equivalents (mmol ACE/g extract).

Table S1. ANN1 model summary (performance and errors), for training, and testing cycles for Yeild

Net. name	Train perf.	Test perf.	Valid perf.	Train error	Test error	Valid error	Training algorithm	Error function	Hidden activation	Output activation
MLP 4-5-1	0.996	0.98 7	0.998	0.058	0.083	0.027	BFGS 167	SOS	Logistic	Identity

Table S2. The weight coefficients and biases W_1 and B_1 for ANN1.

	1	2	3	4	5
C	-1.578	-1.455	-4.045	- 11.013	-1.070
t	-1.804	0.837	1.481	-1.925	-3.251
T	4.937	0.349	-4,240	5.669	-4.428
Ratio	-1.092	-1.603	4.229	0.552	-0.389
Bias	5.422	-2.432	5.694	8.065	4.864

Table S3. The weight coefficients and biases W_2 and B_2 for ANN1.

	1	2	3	4	5	Bias
Yeild	-3.085	-3.47	0.651	0.644	-0.466	2.891

Table S4. ANN2 model summary (performance and errors), for training, and testing cycles for total phenolic

Net. name	Train perf.	Test perf.	Valid perf.	Train error	Test error	Valid error	Train algori t	Error function	Hidden activation	Output activatio n
MLP 4-6-1	0.900	0.977	0.993	2.437	0.338	1.724	BFGS 51	SOS	Exponentia l	Logistic

Table S5. The weight coefficients and biases W_1 and B_1 for ANN2.

	1	2	3	4	5	6
C	0.462	4.716	1.084	0.388	-3.932	3.683
t	2.123	-0.686	1.607	-5.298	-0.639	-3.312
T	1.579	-0.870	-2,893	0.805	2.160	2.285
Ratio	-2.542	-5.450	-2.659	-4.445	-6.662	-6.697
Bias	-2.649	-0.333	-0.458	1.079	2.255	-0.399

Table S6. The weight coefficients and biases W_2 and B_2 for ANN2.

	1	2	3	4	5	6	Bias
Total phenolic	-0.741	0.726	-2.023	1.896	1.409	-1.574	-0.196

Table S7. ANN3 model summary (performance and errors), for training, and testing cycles for total flavonoid

Net. name	Train perf.	Test perf.	Valid perf.	Train error	Test error	Valid error	Train algorithm	Error function	Hidden activation	Output activation
MLP 4-8-1	0.812	0.806	0.848	23.067	18.770	18.236	BFGS 22	SOS	Exponential	Identity

Table S8. The weight coefficients and biases W_1 and B_1 for ANN3.

	1	2	3	4	5	6	7	8
C	2.215	0.120	0.432	0.976	0.039	0.368	-1.169	1.225
t	-0.964	0.439	-0.075	-0.349	0.319	-0.139	0.954	-0.384
T	-0,57	-0.425	-0.196	-0.483	-0.238	-0.389	0.136	-0.348
Ratio	-0.267	0.734	-0.394	0.284	0.953	0.272	0.486	-0.067
Bias	0.062	-0.018	0.010	0.049	-0.077	0.113	-0.566	0.280

Table S9. The weight coefficients and biases W_2 and B_2 for ANN3.

	1	2	3	4	5	6	7	8	Bias
Total flavonoid	-0.317	-0.246	-0.432	0.529	-0.276	0.215	0.407	0.645	-0.062

Table S10. ANN4 model summary (performance and errors), for training, and testing cycles for DPPH

Net. name	Train perf.	Test perf.	Valid perf.	Train error	Test error	Valid error	Train algorithm	Error function	Hidden activation	Output activation
MLP 4-7-1	0.889	0.922	0.985	32.434	30.856	3.086	BFGS 56	SOS	Exponential	Logistic

Table S11. The weight coefficients and biases W_1 and B_1 for ANN4

	1	2	3	4	5	6	7
C	1.602	-0.617	-0.492	0.972	-3.339	-0.537	-3.160
t	-0.206	-0.163	1.342	-0.426	-1.917	-2.269	-0.809
T	-1.200	0.282	0.446	-0.114	3.758	3.495	2.428
Ratio	-0.821	1.203	0.331	0.074	1.946	1.119	4.622
Bias	-0.067	-0.093	-0.453	-0.014	0.963	1.364	-1.428

Table S12. The weight coefficients and biases W_2 and B_2 for ANN4.

	1	2	3	4	5	6	7	Bias
DPPH	1.083	1.108	-0.300	0.183	0.423	-0.119	-0.366	-1.387

Table S13. ANN5 model summary (performance and errors), for training, and testing cycles for ABTS

Net. name	Train perf.	Test perf.	Valid perf.	Train error	Test error	Valid error	Training algorithm	Error function	Hidden activation	Output activation
MLP 4-6-1	0.941	0.850	0.994	29.487	79.370	3.154	BFGS 126	SOS	Exponential	Exponential

Table S14. The weight coefficients and biases W_1 and B_1 for ANN5

	1	2	3	4	5	6
C	-0.892	5.564	0.661	2.038	2.388	-4.213
t	0.745	-1.582	-1.367	-2.757	-4.272	2.156
T	1.114	4.762	-0.451	1.026	-2.476	-2.447
Ratio	1.681	2.618	-1.155	3.878	-4.137	7.163
Bias	-0.462	-4.262	0.938	-1.123	1.853	-1.590

Table S15. The weight coefficients and biases W_2 and B_2 for ANN5.

	1	2	3	4	5	6	Bias
ABTS	0.195	-0.005	1.342	-0.011	-0.559	-0.008	-1.875

Table S16. ANN6 model summary (performance and errors), for training, and testing cycles for CUPRAC

Net. name	Train perf.	Test perf.	Valid perf.	Train error	Test error	Valid error	Train algorithm	Error function	Hidden activation	Output activation
MLP 4-10-1	0.836	0.752	0.817	50.434	100.834	52.903	BFGS 148	SOS	Tanh	Exponential

Table S17. The weight coefficients and biases W_1 and B_1 for ANN6

	1	2	3	4	5	6	7	8	9	10
C	-1.472	1.546	-1.178	-1.140	1.393	-0.604	-6.021	-0.358	-0.466	-0.604

t	-2.579	-1.017	5.205	0.653	-6.401	-4.669	-1.915	2.055	-1.191	2.912
T	2.958	0.392	2.706	3.477	5.578	8,408	8.040	0.157	0.680	0.560
Ratio	-1.440	-2.876	5.052	4.337	-2.653	5.967	7.933	-0.209	0.886	1.826
Bias	-1.435	1.387	-1.159	1.187	3.902	-6.959	-2.972	-0.615	-0.783	1.254

Table S18. The weight coefficients and biases W_2 and B_2 for ANN6.

	1	2	3	4	5	6	7	8	9	10
CUPRAC	4.257	-2.956	4.066	-2.193	2.347	-1.898	-1.107	-0.323	0.123	1.335

Table S19. ANN7 model summary (performance and errors), for training, and testing cycles for FRAP

Net. name	Train perf.	Test perf.	Valid perf.	Train error	Test error	Valid error	Trainalgorithm	Error function	Hidden activation	Output activation
MLP 4-4-1	0.634	0.682	0.994	89.654	124.955	158.057	BFGS 19	SOS	Logistic	Logistic

Table S20. The weight coefficients and biases W_1 and B_1 for ANN7

	1	2	3	4
C	-17.187	0.305	-10.415	-7.861
t	-2.343	0.339	-1.579	-1.155
T	3.389	-2.475	10.312	3.539
Ratio	-12.265	-0.192	-5.997	-5.403
Bias	-0.620	0.232	-2.684	-0.877

Table S21. The weight coefficients and biases W_2 and B_2 for ANN7.

	1	2	3	4	Bias
FRAP	2.695	1.451	2.714	-0.267	-0.507

Table S22. ANN8 model summary (performance and errors), for training, and testing cycles for α-amylase

Net. name	Train perf.	Test perf.	Valid perf.	Train error	Test error	Valid error	Train algorithm	Error function	Hidden activation	Output activation
MLP 4-8-1	0.808	0.812	0.933	0.001	0.001	0.001	BFGS 91	SOS	Exponential	Exponential

Table S23. The weight coefficients and biases W_1 and B_1 for ANN8

	1	2	3	4	5	6	7	8
C	-4.089	-0.720	1.795	4.750	0.120	0.136	7.297	3.820
t	0.051	-0.303	0.048	0.041	-0.268	-0.173	0.033	-0.077
T	-0.131	0.415	0.712	-0.197	0.403	-0.065	-0.214	-0.720
Ratio	0.123	-0.213	-0.138	0.002	-0.114	-0.178	-0.077	-0.877
Bias	0.313	-0.179	0.463	0.348	-0.835	-0.549	-1.463	0.685

Table S24. The weight coefficients and biases W_2 and B_2 for ANN8.

	1	2	3	4	5	6	7	8	Bias
α-amylase	-3.311	-1.755	-0.116	-0.387	-1.168	-0.652	0.196	-0.099	6.570

Table S25. ANN9 model summary (performance and errors), for training, and testing cycles for Tyrosinase

Net. name	Train perf.	Test perf.	Valid perf.	Train error	Test error	Valid error	Train algorithm	Error function	Hidden activation	Output activation
MLP 4-6-1	0.813	0.767	0.965	0.265	0.523	0.117	BFGS 41	SOS	Logistic	Logistic

Table S26. The weight coefficients and biases W_1 and B_1 for ANN9

	1	2	3	4	5	6
C	1.587	5,166	-0.418	0.672	-0.154	2.104
t	-0.295	-3.362	0.694	-0.003	0.924	-0.629
T	10.716	7.683	-1.636	1.497	2.052	2.454
Ratio	-6.618	-0.791	0.691	-1.349	-1.924	-1.786
Bias	-0.430	2.217	1.972	-0.066	-0.148	-0.921

Table S27. The weight coefficients and biases W_2 and B_2 for ANN9.

	1	2	3	4	5	6	Bias
Tyrosinase	-3.984	3.173	-2.639	1.274	1.644	2.042	-0.888

Table S28. The "goodness of fit" for the observed ANN models

	χ^2	RMSE	MBE	MPE	r^2
Yeild	0.017	0.128	0.008	0.197	0.994
TPC	0.365	0.596	0.016	0.194	0.948
TFC	4.685	2.136	0.078	1.185	0.779
DPPH	5.007	2.208	-0.101	0.163	0.929
ABTS	4.302	2.047	-0.144	0.131	0.972
CUPRAC	3.541	1.857	0.221	0.149	0.944
FRAP	36.884	5.994	-0.364	0.541	0.558
α-amylase	0.000	0.013	0.004	0.814	0.891
Tyrosinase	0.159	0.393	0.102	0.189	0.694
